# Bragg polarization gratings for wide angular bandwidth and high efficiency at steep deflection angles

**DOI:** 10.1038/s41598-018-25535-0

**Published:** 2018-05-08

**Authors:** Xiao Xiang, Jihwan Kim, Michael J. Escuti

**Affiliations:** 1 Department of Physics, North Carolina State University, Raleigh, North Carolina 27695 USA; 2 Department of Electrical and Computer Engineering, North Carolina State University, Raleigh, North Carolina 27695 USA

## Abstract

Optical films and surfaces using *geometric* phase are increasingly demonstrating unique and sometimes enhanced performance compared to traditional elements employing *propagation* phase. Here, we report on a diffraction grating with wider angular bandwidth and significantly higher average first-order efficiency than the nearest prior art of metasurfaces, volume holographic gratings, and surface-relief gratings configured to achieve a steep deflection angle. More specifically, we demonstrate a liquid crystal (LC) polymer Bragg polarization grating (PG) with large angular bandwidth and high efficiency in transmission-mode for 532 nm wavelength and 400 nm period. Angular bandwidth was significantly increased by arranging two slanted grating layers within the same monolithic film. First, we studied the optical properties with simulation and identified a structure with 48° angular bandwidth and 70% average first-order efficiency. Second, we fabricated a sample using a photo-aligned chiral nematic LC, where the two grating slants were controlled by the chiral dopants. We measured 40° angular bandwidth, 76% average efficiency, and 96% peak efficiency. Strong input polarization sensitivity (300:1 contrast) and spectral bandwidth (200 nm) mostly matched prior PGs. This approach is especially advantageous for augmented-reality systems and nonmechanical beam steering.

## Introduction

Deflecting light to large angles with a thin transmissive optical element is challenging to do with high efficiency for a wide range of incident angles and wavelengths^1^. One approach uses various types of transmission-mode diffraction gratings with periods near or below the wavelength of operation, which are particularly useful in waveguide-based head-mounted-displays^1–3^ and nonmechanical beam-steering^4,5^. Current technologies all present undesirable trade-offs between efficiency, angular/spectral bandwidths, and manufacturing viability. In this paper, we demonstrate a Bragg polarization grating with sub-wavelength period and significantly increased angular bandwidth, in both simulation and experiment. This element maintains high first-order efficiency, wide spectral bandwidth, and strong polarization sensitivity.

Several transmissive gratings can manifest near-100% diffraction efficiency near the Bragg condition. However, their angular and spectral bandwidths vary greatly, defined here as the angles near normal incidence over which efficiency *η* ≥ 30% and the wavelengths over which *η* ≥ *η*_*max*_/2 (*i*.*e*., full-width-half-maximum), respectively. Volume holographic gratings (VHGs)^6–8^ usually manifest angular and spectral bandwidths of ≤10° and ≤100 nm, while wider bandwidths may be accomplished by superposition within the same film^7–9^. Surface-relief gratings (SRGs)^10^ in general have wider bandwidths^11,12^, as high as 25° and 100 nm, but cannot be easily superimposed like VHGs.

Two recent reports on nontraditional technologies are also notable. First, a study^1^ on a Si-nanobeam metasurface with period Λ = 380 nm demonstrated an experimental angular bandwidth of about 20° (under our definition) and about 40° (under their more relaxed definition), and implied a spectral bandwidth of 200 nm. Most notably, however, the average efficiency demonstrated experimentally was only about 30%. Second, we reported^13^ on a Bragg polarization grating (PG) at Λ = 335 nm with bandwidths of 20° and 200 nm, and an average efficiency within the bandwidth of about 55%. This film had a single-slant grating consisting of a photo-aligned^14^ liquid crystal polymer (LCP) network^15^ (also known as reactive mesogens), fabricated by coating multiple sublayers^16,17^ on a single alignment surface. One important distinction amongst the four grating technologies cited above relates to their fundamental principle of diffraction: Bragg PGs function via the *geometric* phase, while the others (VHGs, SRGs, and nanobeam metasurfaces) function via the *propagation* phase^18^. The former manifests unique general behavior distinct from the latter, including stronger input polarization sensitivity and high single-order efficiency with often lower higher-order leakages.

## Results

### Principles of expanding angular bandwidth in Bragg PGs

In this work, we aim to increase the angular response of Bragg PGs by creating two or more slants within the same physical grating, similar to VHGs. While we cannot superimpose gratings within the same volume, we can easily layer them because the last LCP surface spontaneously aligns subsequent layers. Two essential features of LC polymer Bragg PGs^13^ make this possible: (1) the grating slant is controlled by the concentration of a chiral dopant within the LC prepolymer mixture, and (2) the direction of diffracted light is determined entirely by the surface grating period and is not affected by the slant angle. Here, we design and fabricate a Bragg PG with two slants, which we use to demonstrate a significantly larger angular bandwidth without sacrificing efficiency or spectrum.

**Figure 1:**
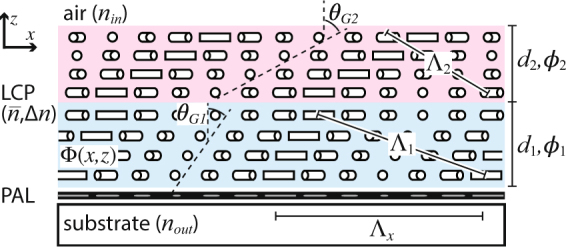
Illustration of the director profile of the two-slant LC polymer Bragg PG. All notation follows ref.^13^.

In general, the in-plane orientation angle Φ of a PG^13,19^ may vary both along the surface *x* and the thickness *z*, when a chiral nematic material is employed. For a set of multiple twisted nematic layers, as in Fig. 1, the director follows:

1 $${{\rm{\Phi }}}_{i}(x,z)=\pi x/{{\rm{\Lambda }}}_{x}+{\varphi }_{i}z/{d}_{i}+{{\rm{\Phi }}}_{0i},$$

where *ϕ*_*i*_ and *d*_*i*_ are the twist angle and thickness of the *i*th layer, respectively, Λ_*x*_ is the surface period, and Φ_0*i*_ is the relative offset accounting for the twists of lower layers. As mentioned in our previous work^13^, the twist rate *ϕ*_*i*_/*d*_*i*_ determines the slant angle *θ*_*Gi*_ via

2 $$\tan \,{\theta }_{Gi}={{\rm{\varphi }}}_{i}{{\rm{\Lambda }}}_{x}/{d}_{i}\pi .$$

The twist rate therefore also controls the peak efficiency angle *θ*_*Pi*_, as measured in the incident medium *n*_*in*_:

3 $$\sin \,{\theta }_{Pi}=(\bar{n}/{n}_{in})\,\sin \,({\theta }_{Bi}+{\theta }_{Gi}),$$

where the Bragg angle is defined as $|\sin \,{\theta }_{Bi}|=\lambda \mathrm{/(2}\bar{n}{{\rm{\Lambda }}}_{i})$, and where $\bar{n}$ and *λ* are the average index of the layer and the vacuum wavelength, respectively. Note that Λ_*i*_ = Λ_*x*_ cos *θ*_*Gi*_ is the volumetric grating period. Note that this independence of the diffraction direction on the slant angle (*i*.*e*., Eq. (5) in ref.^13^) is different than the case of slanted VHGs, which record Λ_*i*_ instead of Λ_*x*_.

Therefore, it stands to reason that a two-slant structure (Fig. 1) should enable wider angular bandwidth, when a first slant has a smaller *θ*_*P*1_ (created by a smaller *θ*_*G*1_ and *ϕ*_1_/*d*_1_) and a second slant has larger *θ*_*P*2_. Based on the one-slant result^13^, we anticipate that the peak angles should be near ±10° if we aim for a bandwidth of 40°.

### Design and Simulation

To obtain an optimal solution, we simulated a two-slant Bragg PG structure using rigorous-coupled-wave-analysis^20^, with Λ = 400 nm, *λ* = 532 nm, Δ*n* = 0.25, *n*_*in*_ = 1, $\bar{n}=1.65$, *n*_*out*_ = 1.7, and left-hand-circular (LHC) input polarization. Using Matlab optimization tools and a multistart method, we searched for a global minimum of the merit function $f={\mathtt{mean}}({(100 \% -{\eta }_{-1}({\theta }_{in}))}^{2})$, where *η*_−1_(*θ*_*in*_) is the first-order efficiency as a function of input angle of incidence, aiming to find the highest average efficiency across ±20°, *i*.*e*., an angular bandwidth of 40°. The optimal solution was (*ϕ*_1_, *d*_1_) = (−120°, 0.83 *μ*m) and (*ϕ*_2_, *d*_2_) = (−253°, 0.66 *μ*m).

**Figure 2:**
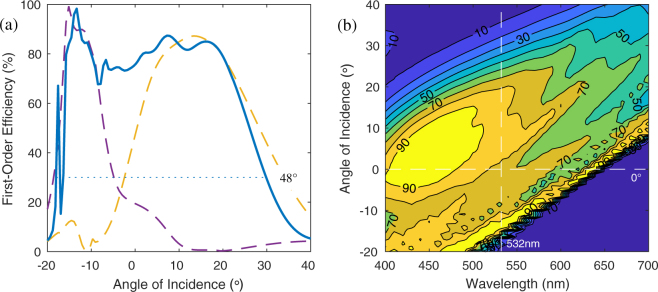
Simulated first-order efficiency *η*_−1_ of the optimized two-slant LC polymer Bragg PG: (**a**) angular response at *λ* = 532 nm of both slants (blue solid) acting as the unit as illustrated in Fig. 1, as well as the first (gold) and second (purple) slants individually; and (**b**) angular and spectral response.

The simulated angular and spectral response of the two-slant Bragg PG is shown in Fig. 2 for LHC input polarization, with a predicted angular bandwidth 48° at 532 nm and an average efficiency therein of about 70%. At normal incidence, the spectral bandwidth is at least 200 nm. The angular response of the individual slants is shown in Fig. 2(a), with peaks angles at about ±13°. The Eq. 3 predicts slant angles of *θ*_*G*1_ = −17.8° and *θ*_*G*2_ = −40.5°. Note that the simulated efficiency for right-hand-circular (RHC) polarization in this same range is ≤0.1%.

### Experiment

**Figure 3:**
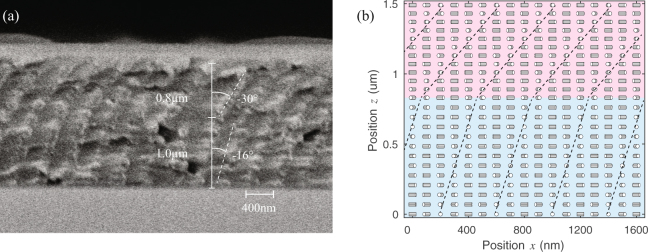
Structure of the two-slant Bragg PG: (**a**) SEM micrograph of fabricated sample, and (**b**) calculated director profile of the optimal solution (shown with equal XY aspect ratio). Dashed lines indicate iso-orientation “planes” within each period, corresponding to the each sublayer slant.

After fabrication, we examined the nanoscale nematic director profile with a scanning electron microscope (SEM). We first submersed it in liquid nitrogen, then broke it, and finally evaporated a 5 nm layer of gold onto it. The cross-section of the edge is shown in Fig. 3(a), where a monolithic grating with two different slant angles can be clearly observed. This same grating texture was observed over the entire 20 mm wide exposure area. For comparison, we illustrate the ideal director profile of the optimal solution using Eq. (1) in Fig. 3(b).

We measured optical properties with an out-coupling prism and index matching oil, as detailed previously^13^, most specifically the setup illustrated there in Fig. 2. We define efficiency and transmittance as *η*_*m*_ = *P*_*m*_/(*P*_−1_ + *P*_0_) and *T*_*m*_ = *P*_*m*_/*P*_*in*_ respectively, where *P*_*m*_ is the output power of order *m* and *P*_*in*_ is the input power. These are nearly the same in our case, because the primary loss at *λ* = 532 nm is the reflections at air-glass interfaces (about 5% each).

**Figure 4:**
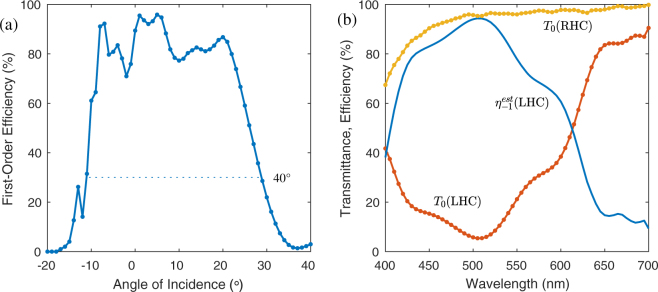
Measured (**a**) angular response for LHC input polarization at *λ* = 532 nm, and (**b**) spectral response for circular input polarization at normal incidence.

The measured angular response is shown in Fig. 4(a), obtained using a 532 nm laser polarized to LHC and using the angle convention of our prior work^13^. The angular bandwidth was 40°, *i*.*e*., −11° to 29°. The average efficiency within these angles was 76%, with a maximum around 96% near normal incidence.

The measured spectral response is shown in Fig. 4(b), for LHC and RHC input polarizations, obtained using a spectrophotometer. As expected, the zero-order transmittance *T*_0_ for LHC input was below 50% for at least 400–600 nm, which is the bandwidth of first-order diffraction. However, when the input was RHC, the first-order diffraction was near zero, and the film is essentially transparent. The measured value of *T*_0_ for RHC departs from nearly 100% primary at deep-blue wavelengths, due to absorption of the film and substrates. We estimate first-order efficiency as ${\eta }_{-1}^{est}=({T}_{0}(RHC)-{T}_{0}(LHC))/{T}_{0}(RHC)$, which corresponds well to the simulation (Fig. 2(b)) and laser measurement (Fig. 4(a)).

**Figure 5:**
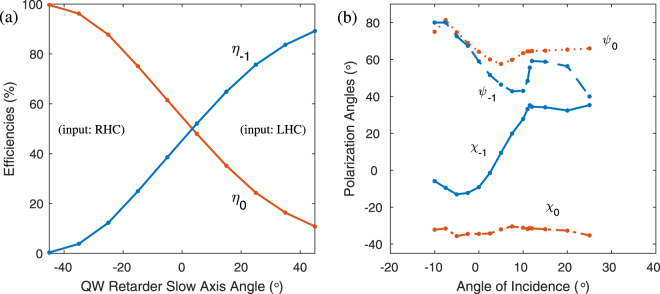
Measured polarization response: (**a**) efficiencies as input polarization is varied by rotating a quarterwave (QW), and (**b**) output polarization angles (orientation *ψ*, ellipticity *χ*) of the first- and zero-orders.

Two polarization measurements were performed. First, we measured the zero- and first-order efficiencies as the incident polarization was varied by rotating a quartz quarterwave (QW) plate receiving linearly polarized light, to vary the input from circular to linear and back to the orthogonal circular polarization. The result is shown in Fig. 5(a), which matches the traditional behavior of all other PGs including both Raman-Nath^21^ and Bragg PGs^13^. The highest and lowest efficiencies occur for LHC and RHC, respectively. The extinction ratio *η*_−1_ : *η*_0_ was about 300:1, which confirms that for RHC input this two-slant Bragg PG is nearly transparent.

Second, we measured the output polarization for LHC input across the angular bandwidth. The result is shown in Fig. 5(b). Most notably, the first-order output polarization is nearly circular for larger angles ≥12° and nearly linear for smaller angles ≤5°. Note that the handedness of the first-order (right) is generally opposite that of the input (left). Conversely, the polarization of the zero-order nearly matches the input.

## Discussion

The concept introduced here uses two Bragg PG layers with different slants and distinct angular responses (Fig. 2(a)), which add up together to support an extremely wide angular bandwidth. Overall, the experimental results match simulation well, except for very negative angles (≤−11°). Of course, this may relate to the differences between the ideal and actually realized values of Δ*n*, $\bar{n}$, *d*_*i*_, and *ϕ*_*i*_. For example, in the SEM image we observe *d*_*i*_ and *θ*_*Gi*_ values that are different than the ideal, especially *θ*_*G*2_. Another difference is the presence of a perfect anti-reflection-coating in simulation that is absent in experiment. We speculate that higher efficiency at more negative angles is possible if the fabricated sample was made to have a larger *θ*_*G*2_.

While only two distinct layers were explicitly studied here, this approach is much more general. It is possible to use three or more slants, *e*.*g*., to enable even wider angular bandwidths and achieve angular response shapes different than a flat-top. In the extreme, the chiral concentration of each sublayer could be slightly different so that the slant angle is effectively smoothly varying throughout the thickness, *i*.*e*., chirped. Another possibility is to achieve a wide angular response in combination with a narrow spectral response. This should be possible if an LC with much lower Δ*n* was employed instead. Multi-slant LC polymer Bragg PGs offer several novel opportunities to realize angular and spectral behavior that may be unfeasible with VHGs, SRGs, or metasurfaces.

As with one-slant Bragg PGs^13^, the output polarization response of a two-slant Bragg PG departs noticeably from circular for many angles. We observe this in both simulation and experiment, and must be related to the fact that the first-order wave propagates at large angles (35° to 80° for ±20° incidence in air) within the birefringent LC layer itself. It should therefore be possible to apply the principles of retardation compensation to adjust the output polarization for a particular application.

**Figure 6:**
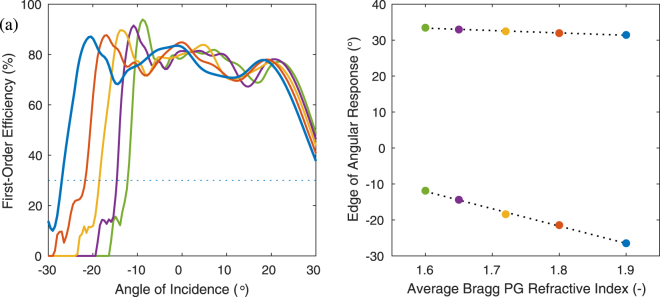
Effect of average Bragg PG refractive index $\bar{n}$: (**a**) Simulated angular response and (**b**) corresponding edges for two-slant design optimized for $\bar{n}=1.9$ (blue curve and dots). The other colors (red, gold, purple, and green) correspond to lower values of $\bar{n}$ (1.8, 1.72, 1.65, and 1.6), respectively. Simulation parameters are otherwise the same as Fig. 2(a).

We notice in both simulation and experiment that the angular response tends to be asymmetric due to a sharp cut-off at negative angles. Note that in our convention, the diffracted (*m* = −1) wave propagates very obliquely within the grating at these incident angles, leading to a strong interaction with the Bragg PG layer interfaces. Eventually, this first-order wave becomes evanescent. While we cannot here discuss exhaustively the conditions required to achieve a maximum angular bandwidth or symmetric angular response, we can at least address the most important point: the average index $\bar{n}$ of the Bragg PG strongly influences the negative angle cut-off. Consider the two-slant grating defined by (*ϕ*_1_, *d*_1_) = (−100°, 0.84 *μ*m) and (*ϕ*_2_, *d*_2_) = (−253°, 0.78 *μ*m), whose angular response is shown in Fig. 6(a). When the average index of the Bragg PG is highest ($\bar{n}=1.9$), its angular bandwidth is approximately ±30°. However, as $\bar{n}$ decreases, the lower edge of the angular response becomes more limited (*i*.*e*., more positive). The upper edge remains relatively unaffected. For simplicity and to isolate the impact of $\bar{n}$, we assumed the output medium index ${n}_{3}=\bar{n}$ for these simulations. These lower and upper edges are summarized in Fig. 6(b). The primary lesson is that a higher average index of the Bragg PG leads to a wider and more symmetric angular response, for a fixed Bragg PG design.

During the final preparation of this manuscript, a reflective version of a single slant polarization grating was published in ref.^19^ with an angular and spectral bandwidths about 20° and 50 nm respectively. In that work, different chiral concentrations were used to expand the inherently modest spectral bandwidth rather than the angular bandwidth. It may be possible to apply the principle we demonstrate here to reflective-mode LC polymer Bragg PGs.

In conclusion, we have shown that the multi-slant Bragg grating studied here in simulation and experiment manifest a large angular bandwidth while maintaining high efficiency and large spectral bandwidth. This was achieved by layering two slants within the same LC polymer Bragg PG monolithic film. Specifically, this grating with 400 nm period measured at 532 nm wavelength manifest in experiment a 40° angular bandwidth, 200 nm spectral bandwidth, 76% average efficiency, and 96% peak efficiency. Similar to the prior one-slant nanoscale Bragg PG work^13^, it was strongly selective to input polarization (300:1 contrast), and the output polarization of the first-order varied from linear to circular, depending on incidence angle.

These results represent the widest angular bandwidth ever demonstrated for diffraction gratings in a comparable configuration, including VHGs, SRGs, and metasurfaces^1^. The set of Λ and *λ* selected here lead to an angle of about −51° inside a high index (*n*_*out*_ = 1.7) substrate for normal incidence, which is relevant to waveguide-based head-mounted-displays^1–3^. Because two-slant Bragg PGs can manifest an angular bandwidth twice as wide and an average efficiency more than twice as high as the closest prior art, they are well suited to support the latest architectures of exit-pupil-expanders (*e*.*g*., refs^22–24^) potentially allowing even wider fields-of-view and lower optical loss. Furthermore, these gratings can also be configured for infrared light to achieve elements analogous to efficient blazed gratings and/or waveguide couplers, for optical telecommunications applications.

## Methods

Fabrication followed the process detailed previously^13^ for the photo-alignment layer (PAL), exposure, and first LC sublayer, except that the holographic beams were separated by ±26.3°, the fabrication substrate had a high index (*n*_*out*_ = 1.71), and no endcap was used. The first slant (*ϕ*_1_, *d*_1_) was formed by coating and curing 18 sublayers of the reactive mesogen solution comprising 3.8% nonchiral nematic RMM-B (Merck KGaA, Δ*n* = 0.25 and $\bar{n}=1.65$ @ 532 nm), 0.2% chiral nematic RMM-C (Merck KGaA, helical pitch ~400 nm and $HTP\sim 2.5\,\mu {m}^{-1}$), and 96% solvent propylene-glycol-methyl-ether-acetate (PGMEA from Sigma-Aldrich). The second slant (*ϕ*_2_, *d*_2_) was formed by 14 sublayers of the reactive mesogen solution comprising 3.6% RMM-B, 0.4% RMM-C, and 96% PGMEA. The coating and curing conditions for the two slants were the same as the prior work^13^, except for spin speed 600 rpm spin speed and 60 s cure time.
